# Parameterization to NDDO-based polarizable force field

**DOI:** 10.1186/1758-2946-6-S1-P53

**Published:** 2014-03-11

**Authors:** Heike Thomas, Matthias Hennemann, Stefan Guessregen, Timothy Clark

**Affiliations:** 1 Computer-Chemie-Centrum, Friedrich-Alexander-Universität Erlangen-Nürnberg, Nägelsbachstraße 25, 91052 Erlangen, Germany; 2 Sanofi Deutschland GmbH, R&D, LGCR, Structure, Design and Informatics, Building G878, 65926 Frankfurt am Main, Germany; 3 Centre for Molecular Design, University of Portsmouth, King Henry Building, Portsmouth PO1 2DY, UK

## 

In Computer-Aided-Drug-Design (CADD), the electrostatic interactions contribute strongly to the interaction between the drug-molecule and the target. Further, the Coulomb term is crucial for calculating the electrostatic contribution to the solvation energy. In spite of this, conventional Force Fields use the obsolete physical concept of point-monopoles (net atomic charges) and thus, are not able to represent the molecular electrostatic potential (MEP) accurately or are even wrong for atoms that have positively and negatively charged areas on their surface [1]. A far better way to describe the MEP is the is multipole-based semiempirical MO-theory [2,3]. For the parameterization of the polarizable hpCADD Force Field, the two methods are combined in order to obtain both the MEP and structures and energies. Additionally, the differentiation of atom-types leads to more detailed information about the MEP.
